# Claw morphometrics in monitor lizards: Variable substrate and habitat use correlate to shape diversity within a predator guild

**DOI:** 10.1002/ece3.4185

**Published:** 2018-06-11

**Authors:** Domenic C. D'Amore, Simon Clulow, J. Sean Doody, David Rhind, Colin R. McHenry

**Affiliations:** ^1^ Department of Natural Sciences Daemen College Amherst New York; ^2^ School of Environmental and Life Sciences University of Newcastle Callaghan NSW Australia; ^3^ Department of Biological Sciences Macquarie University Sydney NSW Australia; ^4^ Department of Biological Sciences University of South Florida– St. Petersburg St. Petersburg Florida; ^5^ School of Biological Sciences Monash University Clayton Vic. Australia; ^6^ Department of Anatomy and Developmental Biology Monash University Clayton Vic. Australia; ^7^ School of Engineering University of Newcastle Callaghan NSW Australia

**Keywords:** ecomorphology, niche partitioning, semilandmarks, the Kimberley, Varanidae, Western Australia

## Abstract

Numerous studies investigate morphology in the context of habitat, and lizards have received particular attention. Substrate usage is often reflected in the morphology of characters associated with locomotion, and, as a result, claws have become well‐studied ecomorphological traits linking the two. The Kimberley predator guild of Western Australia consists of 10 sympatric varanid species. The purpose of this study was to quantify claw size and shape in the guild using geometric morphometrics, and determine whether these features correlated with substrate use and habitat. Each species was assigned a Habitat/substrate group based on the substrate their claws interact with in their respective habitat. Claw morphometrics were derived for both wild caught and preserved specimens from museum collections, using a 2D semilandmark analysis. Claw shape significantly separated based on Habitat/substrate group. *Varanus gouldii* and *Varanus panoptes* claws were associated with sprinting and extensive digging. *Varanus mertensi* claws were for shallow excavation. The remaining species’ claws reflected specialization for some form of climbing, and differed based on substrate compliance. *Varanus glauerti* was best adapted for climbing rough sandstone, whereas *Varanus scalaris* and *Varanus tristis* had claws ideal for puncturing wood. Phylogenetic signal also significantly influenced claw shape, with Habitat/substrate group limited to certain clades. Positive size allometry allowed for claws to cope with mass increases, and shape allometry reflected a potential size limit on climbing. Claw morphology may facilitate niche separation within this trophic guild, especially when considered with body size. As these varanids are generalist predators, morphological traits associated with locomotion may be more reliable candidates for detecting niche partitioning than those associated directly with diet.

## 1 INTRODUCTION

Ecomorphology investigates the functional design of an organism in relationship with its environment, as morphology can limit the ability for said organism to obtain resources (Wainwright, 1991). Numerous systems and morphological traits have been explored to determine how morphology links with performance and habitat (Arnold, 1983; Findley & Black, 1983; James, 1982; Karr & James, 1975; Losos, 1990a; Melville & Swain, 2000; Williams, 1972). Lizards have often been study systems to test such principles, with investigations of body proportions (Herrel, Meyers, & Vanhooydonck, 2001; Thompson & Withers, 1997; Vanhooydonck & Van Damme, 1999), clinging, sprinting, and jumping ability (Irschick et al., 1996,2005; Losos, 1990b; Losos & Sinervo, 1989; Van Damme, Aerts, & Vanhooydonck, 1997; Zamora‐Camacho, Reguera, Rubiño‐Hispán, & Moreno‐Rueda, 2014), retreat choice (Thompson, Clemente, Withers, Fry, & Norman, 2009), limb bone loading and gait (Clemente, Withers, Thompson, & Lloyd, 2011; McElroy & Reilly, 2009), and biting structures (Herrel, Spithoven, Van Damme, & De Vree, 1999; Herrel, Van Damme, Vanhooydonck, & Vree, 2001; Verwaijen, Van Damme, & Herrel, 2002).

Morphological adaptations associated with substrate usage are often reflected in locomotor traits (Grizante, Navas, Garland, & Kohlsdorf, 2010; Losos, 1990b; Vanhooydonck, Andronescu, Herrel, & Irschick, 2005). Claws are therefore well studied, as they are often the first and last structure to interface with substrate during locomotion (Birn‐Jeffery, Miller, Naish, Rayfield, & Hone, 2012). Claw characteristics in lizards have been correlated with performance variables such as clinging and sprinting (Crandell, Herrel, Sasa, Losos, & Autumn, 2014; Tulli, Abdala, & Cruz, 2011, 2012; Zani, 2000) or habitat/microhabitat preference (Ribas et al., 2004; Teixeira‐Filho, Rocha‐Barbosa, Paes, Ribas, & de Almeida, 2001; Tulli, Cruz, Herrel, Vanhooydonck, & Abdala, 2009). Bird claws have also received significant attention (Hahn, Dimitrov, Rehse, Yohannes, & Jenni, 2014), focusing on curvature and its relationship with habitat (Bock & Miller, 1959; Fowler, Freedman, & Scannella, 2009; Glen & Bennett, 2007; Mosto & Tambussi, 2014; Pike & Maitland, 2004). Large comparative studies of mammals have used claws/unguals to determine locomotor, and in particular fossorial, adaptations (MacLeod & Rose, 1993). Modern claws are often correlated with those of nonavian dinosaurs to extrapolate paleo‐behavior (Burnham, Feduccia, Martin, & Falk, 2011; Fowler, Freedman, Scannella, & Kambic, 2011; Lautenschlager, 2014). These studies quantified claw morphology in several ways, including Euclidean distance measures, claw curvature based on triangles, outline‐based morphometrics, and digital modelling (respective examples in Ribas et al., 2004; Feduccia, 1993; MacLeod & Rose, 1993; Manning et al., 2009). Most studies of lizard claw morphology in particular are a variation of the method presented by Zani (2000), which combined Euclidean measures and angular values.

Claws may function as ecomorphologically significant traits within trophic guilds, potentially allowing members to exploit different habitats and reduce interspecific competition. Guilds are defined as multiple species that exploit a similar resource in a similar way (Root, 1967). The monitor lizards (Family: Varanidae) of northern Australia form closely related top‐predator guilds (Wilson & Swan, 2013). The Kimberley (Western Australia) guild consists of 10 sympatric varanid species. Guild members are characterized as generalist, opportunistic predators possessing a degree of dietary overlap (Losos & Greene, 1988; Shine, 1986). The spread of the invasive cane toad (*Rhinella marinas*) has decreased populations of many varanid species across northern Australia (Doody et al., 2009; Doody, James, et al., 2014; Doody, Mayes, et al., 2014; Doody et al., 2017; Doody, Soanes, et al., 2015; Shine, 2010), potentially changing the nature of these guilds.

The purpose of this study was to quantify the morphological variability in the claws of the Kimberley monitor lizard guild, and determine whether it correlated with function and ecology. Our major hypothesis was if monitor lizard claws interacted with a variety of substrates, then they would have significantly different claw morphologies. We clustered the varanid species into ecological groups based on the substrate their claws typically interact with within their respective habitats. We then measured both forelimb and hindlimb claws, and analyzed them using geometric morphometrics. We also investigated how allometry and phylogeny might also influence claw structure. Lastly we explored how claw morphology could potentially facilitate niche separation in the Kimberley monitor guild.

## 2 MATERIALS AND METHODS

### 2.1 Ecological assignments

The Kimberley landscape is diverse, with gorges, boulder fields, riparian zones, and savannahs of flat, open grasslands. As habitat, locomotor mode, and substrate are closely linked, varanid claws may interact with number of substrates to varying degrees. Therefore, varanid species were placed in *a priori* groups based on these factors. Estimates of species substrate usage and locomotor mode were taken from the relevant literature and personal observations (Clemente, Thompson, & Withers, 2009; Openshaw & Keogh, 2014; Thompson et al., 2009; Wilson & Swan, 2013). This resulted in five Habitat/substrate groups (Table 1):

**Table 1:** Kimberley varanid species with Habitat/substrate group, clade according to molecular phylogeny, and number of specimens sampled

Species	Habitat/substrate group	Clade	*N*
*Varanus acanthurus*	Rocky‐field	Acanthurus	15
*Varanus glauerti*	Escarpment	Tristis	7
*Varanus glebopalma*	Rocky‐field	Tristis	5
*Varanus gouldii*	Savannah‐burrower	Gouldii	12
*Varanus kingorum*	Rocky‐field	Acanthurus	3
*Varanus mertensi*	Riverbed	Gouldii	14
*Varanus mitchelli*	Arboreal	Tristis	10
*Varanus panoptes*	Savannah‐burrower	Gouldii	7
*Varanus scalaris*	Arboreal	Tristis	18
*Varanus tristis*	Arboreal	Tristis	4

1. *Arboreal* consists of savannah species which are primarily observed climbing trees. *Varanus scalaris* and *Varanus tristis* may be found within grassland trees (Pianka, 2004; Smith, Sweet, & King, 2004; Sweet, 2007). *Varanus mitchelli* frequents mangroves in the riparian zone, and uses branches to launch into rivers (Schultz & Doody, 2004; Shine, 1986).
2. *Escarpment* consists of monitors that climb large, vertical faces of sandstone escarpments, typically within gorges. This group is solely composed of the saxicolous *Varanus glauerti* (Sweet, 2004a).
3. *Riverbed* consists solely of *Varanus mertensi,* which is almost always found in proximity to permanent freshwater (Christian, 2004a; pers. obs.). This species is known to occasionally bask and/or sleep in trees, but is more typically associated with the rocks and soil of the river's edge. It excavates shallow burrows near water, and forages primarily on semi‐ and fully aquatic river prey (Kennett, Christian, & Pritchard, 1993; Losos & Greene, 1988; Mayes, Thompson, & Withers, 2005; Rhind, Jackson, Pezaro, & Doody, 2016).
4. *Rocky‐field* consists of monitors found in rocky open fields, composed of spinifex grasses, small trees, boulders, and outcrops. These species cross open terrain, climb up rocks, and refuge within crevices. This group includes *Varanus acanthurus, Varanus glebopalma,* and *Varanus kingorum* (Dryden, 2004; King, 2004; Sweet, 2004b).
5. *Savannah‐burrower* consists of large, widely foraging, savannah monitors who burrow extensively in soil (Doody, James, et al., 2014). These species are rarely found in trees, and include *Varanus gouldii* and *Varanus panoptes* (Christian, 2004b; Thompson, 1995).

### 2.2 Specimens and data collection

Claw morphometrics were taken from both wild caught and preserved specimens. Wild‐caught specimens were captured at El Questro Wilderness Park, situated in the Kimberley, Western Australia (15°53′42.1″S, 128°7′56.8″E), during the Dry Season. Lizards were caught using a combination of trapline fences equipped with pit‐ and funnel‐traps (similar to Doody, Clulow, et al., 2015), noosing, and hand‐capture. Field researchers would survey the park in teams, and noose specimens when encountered. Specimens were placed in a breathable cloth bag upon capture, taken back to camp, processed, marked, and released the following day in the same place. Dry‐ and ethanol‐preserved specimens were from the Division of Reptiles and Amphibians in the University of Michigan Museum of Zoology (UMMZ). The only specimens in the collection omitted had damage to the distal tissue where the claw erupted, or the claw was broken or visibly worn. All specimens had their snout‐vent length (SVL) taken using measuring tape.

**Figure 1:**
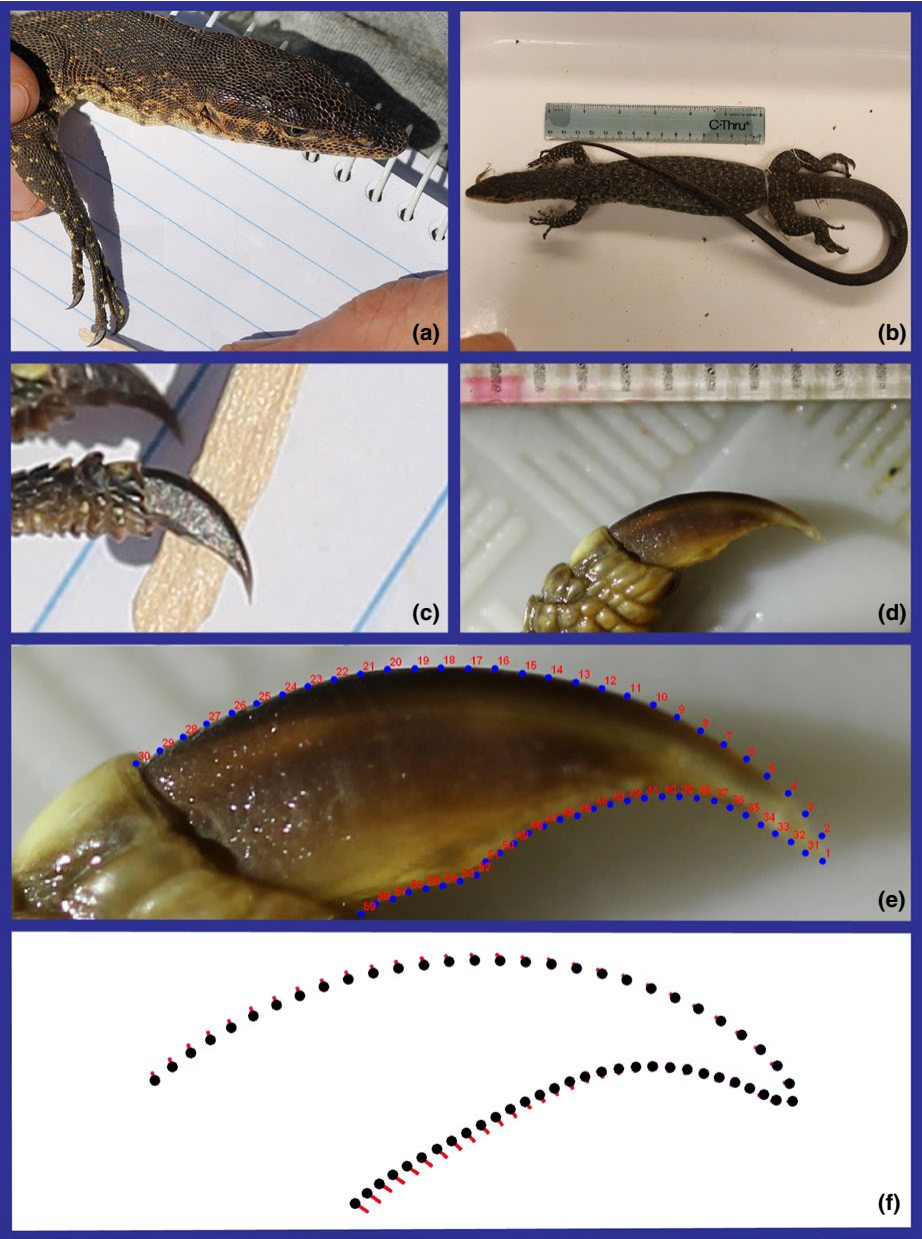
Claws were photographed from both wild caught (a,c; *Varanus mitchelli*, Vmi12) and preserved museum (b,d; *V. mitchelli*, UMMZ 210576) specimens. (e) Fifty‐nine equidistant coordinates were plotted, and semilandmarks were slid using minimum bending energy. (f) Shape variance was represented through vector diagrams

The claw of digit IV of both the forelimb and hindlimb of each side was placed lateral‐side up against a light background with a scale. Photographs were taken with a Canon Rebel T3 EOS and 60 mm Macro lens held perpendicular to the claw. A camera stand ensured proper perspective in the museum. For wild specimens, the lizard was held in position by a researcher while another photographed it (Figure 1).

Tinius and Russell (2017) proposed the use of “pseudolandmarks” (referred to here as semilandmarks) when measuring claws, and we adopted a similar method. This approach, nested in geometric morphometrics (Bookstein, 1997; Zelditch, Swiderski, Sheets, & Fink, 2004), best assumes the totality of claw shape. The margin of the claw was traced from photographs in TpsDig 2.16 using the curve drawing tool (Rohlf, 2010). Tracing started at the base where the claw erupts to the tip, on both the dorsal and palmer/plantar sides (Figure 1). The base of the claw itself was not traced, because (a) our method as is accurately depicted the height of the claw at the base and (b) differences in distal scale morphology would add shape variance that is not relevant to claw function. The two traced margins were transformed into 30 equidistant coordinates, and the coordinates at the tip were combined into one. This resulted in three landmarks and 56 semilandmarks, the latter of which were slid to minimize the bending energy (Gunz & Mitteroecker, 2013; Perez, Bernal, & Gonzalez, 2006) using TpsRelw 1.53 (Rohlf, 2013). This program also performed a generalized least squares Procrustes superimposition on the data, and calculated centroid size (CS). CS is the square root of the sum‐squared distances from the landmarks to the centroid (Zelditch et al., 2004). It is technically a linear measurement, but measures overall size as opposed to a single Euclidean dimension. Bilateral symmetry was assumed; the superimposed coordinates and CS were averaged between left and right sides.

### 2.3 Ordination and statistics

All analyses were conducted in MorphoJ (Klingenberg, 2011) and SPSS Version 19.0 (IBM Corp, Armonk, NY). A 10,000 permutations test of the Procrustes distance between forelimb and hindlimb claws determined there was no significant difference between them (*p *=* *0.0514). Forelimb and hindlimb claws were therefore analyzed together from here on.

Numerous genetic phylogenies of Varanidae exist (Ast, 2001; Clemente et al., 2009; Fitch, Goodman, & Donnellan, 2006; Vidal et al., 2012), and the consensus is that four major clades are present in Australia; the pygmy monitors (“Odatria” clade), the sand monitors (“Gouldii” clade), the lace monitors (“Varius” clade), and the mangrove monitors (“Indicus” clade). Odatria may be further divided into the “Tristis” and “Acanthurus” clades (Table 1). Claw morphometrics were mapped onto a molecular phylogeny with branch lengths in MorphoJ (based on Thompson et al., 2009), to evaluate shape and Habitat/substrate group in reference to relatedness. A 10,000 permutations test against the null hypothesis indicated that phylogenetic signal did influence claw shape (*p *=* *0.0034), so this relationship was investigated further. MorphoJ generated phylogenetically independent contrasts (PICs) as a way of measuring relative shape change decoupled from relatedness (Felsenstein, 1985; Klingenberg & Marugán‐Lobón, 2013). The output was in *x‐y* coordinates for each branching on the phylogeny. These coordinates were converted to Procrustes distances, and a greater value indicated a greater relative shape change decoupled from phylogenetic history. Sample claws and line drawings of species means were also plotted on the phylogeny for qualitative visual comparison.

Allometric reduced major axis regressions (sensu Clarke, 1980) and their residuals were produced using PAST (Hammer, Harper, & Ryan, 2001), plotting CS and shape coefficients against SVL for all individuals. Allometry concerning CS was defined by a statistically significant (*p *<* *0.05) slope deviating from 1. Consistency in shape indicates isometry; therefore, allometric shape change is any significant slope. Residuals were then analyzed to determine significant differences between Habitat/substrate groups once the data were normalized. These residuals were not normally distributed within certain groups according to Shapiro–Wilk tests, but homoscedastic according to Levene's test. Therefore, they were analyzed using a nonparametric analysis of variance (Kruskal–Wallis).

Procrustes distances were calculated between Habitat/substrate groups to determine the pairwise differences in mean shapes. The statistical significance of these distances was assessed with 10,000 permutations tests. A principal components analysis (PCA) was conducted to determine the degree of shape variance within the data set. All PCs representing over 10% of the variance were considered, and were plotted as *x–y* scatter‐based morphospaces. The aforementioned phylogeny was also plotted on the same morphospace resulting in a phylomorphospace.

## 3 RESULTS

### 3.1 Phylogenetic context of Habitat/substrate groups

**Figure 2:**
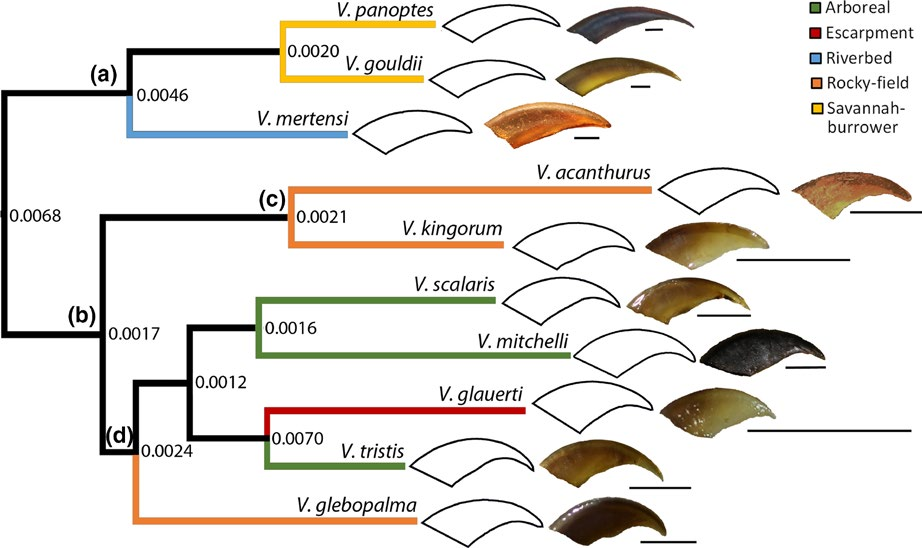
Phylogenetic tree topology of *Varanus* used in this study, based on Thompson et al. (2009) and generated using Mesquite (Maddison & Maddison, 2017). Major clades are labelled, including (a) Gouldii, (b) Odatria, (c) Acanthurus, and (d) Tristis. Numbers at each node indicate phylogenetically independent contrasts in the form of Procrustes distances for associated branches. Line diagrams indicate the mean shape for each species, and colored branches indicate Habitat/substrate group. Scale = 2 mm

Habitat/substrate groups were exclusive to either the Odatria or Gouldii clades, a separation reflected by a relatively large PIC at the root (Figure 2). All Riverbed and Savannah‐burrower taxa were exclusive to Gouldii, as another large PIC signified the separation between these two groups. Although Odatria consisted of three Habitat/substrate groups, the clade mostly showed low PICs with one exception. The entirety of the Acanthurus clade was Rocky‐field with a relatively low PIC. The Tristis clade had one Rocky‐field representative branching off early, with the remaining members mostly being Arboreal. The branching off of the Escarpment taxon from the Arboreal resulted in the largest PIC in our data set.

### 3.2 Allometry

**Figure 3:**
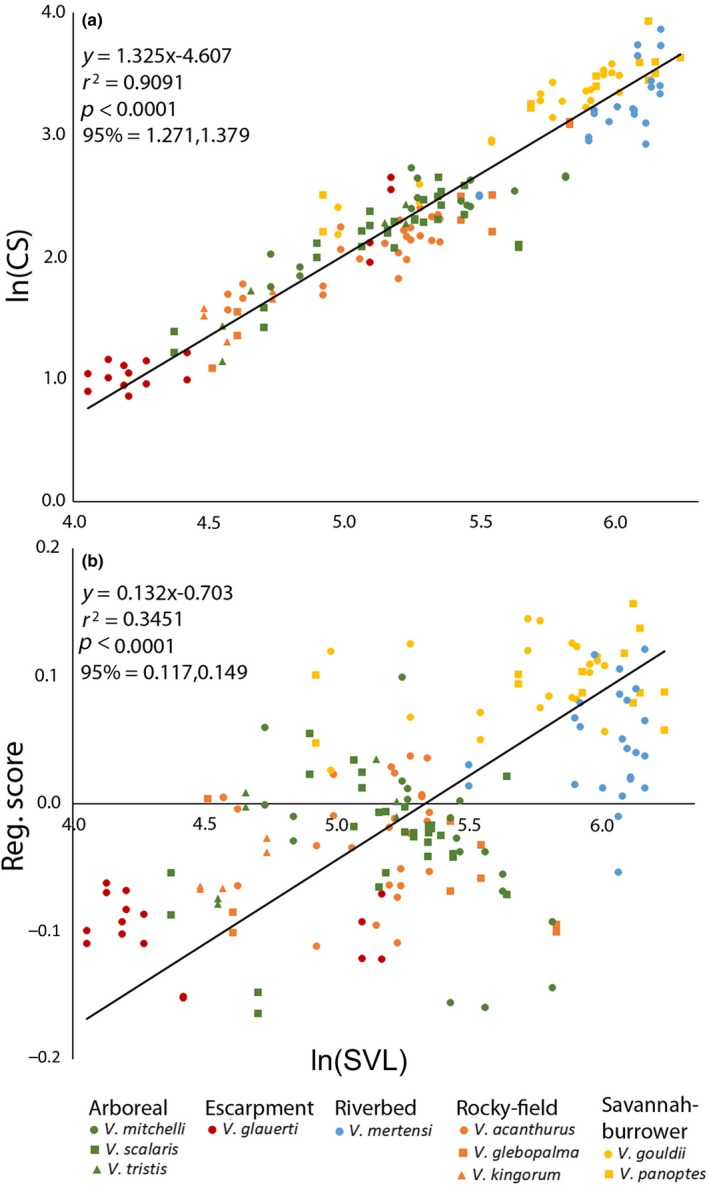
Regression of (a) ln claw centroid size (CS) in millimeters and (b) Shape Coefficients (Reg. Score) versus ln individual Snout‐vent length (SVL) in millimeters for all varanid claws, with regression information and statistics. Habitat/substrate group and species are distinguished by color and shape, respectively

Arboreal, Escarpment, and Rocky‐field taxa typically had smaller SVLs. Claw CS and SVL were highly correlated (*r* ^2^ = 0.91), and indicated positive size allometry regardless of species or Habitat/substrate group (Figure 3). Positive shape allometry, although significant, had a much poorer goodness of fit (*r* ^2^ = 0.35), as shape appeared to be consistent within species regardless of body size (for example, both a 195.7 mm and 385.6 mm SVL *V. gouldii* had shape‐coefficents of ~0.12). This resulted in individuals not conforming closely to the regression. For both size and shape residuals, Savannah‐burrower varanids displayed primarily positive values and Riverbed taxa were mostly negative, with other groups plotting on both sides of the regression. This resulted in a significant difference as indicated by Kruskal–Wallis (Table 2).

**Table 2:** Output of Kruskal–Wallis test of allometric regression residuals between Habitat/substrat*e* groups

Variable	*df*	*χ* ^2^	*p*
Size	4	44.558	<0.0001
Shape	4	26.667	<0.0001

Notes

Regressions plotted in Figure 3.

### 3.3 Shape variability

**Figure 4:**
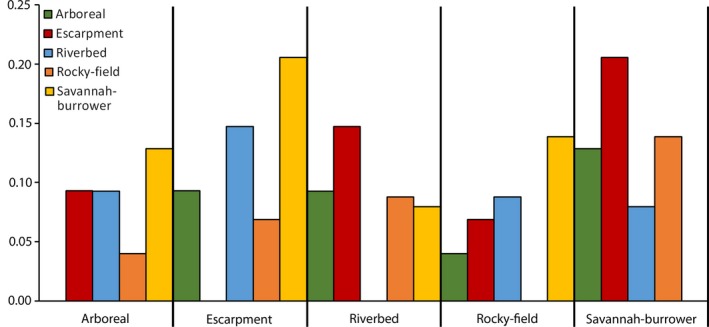
Procrustes distances among Habitat/substrate groups for all claws. All were significant (*p *<* *0.05)

All Procrustes distances between Habitat/substrate groups were statistically significant (Figure 4). Savannah‐burrower was closest to Riverbed, and both were farthest from Escarpment. Arboreal and Rocky‐field were the closest to one another out of all groups.

**Figure 5:**
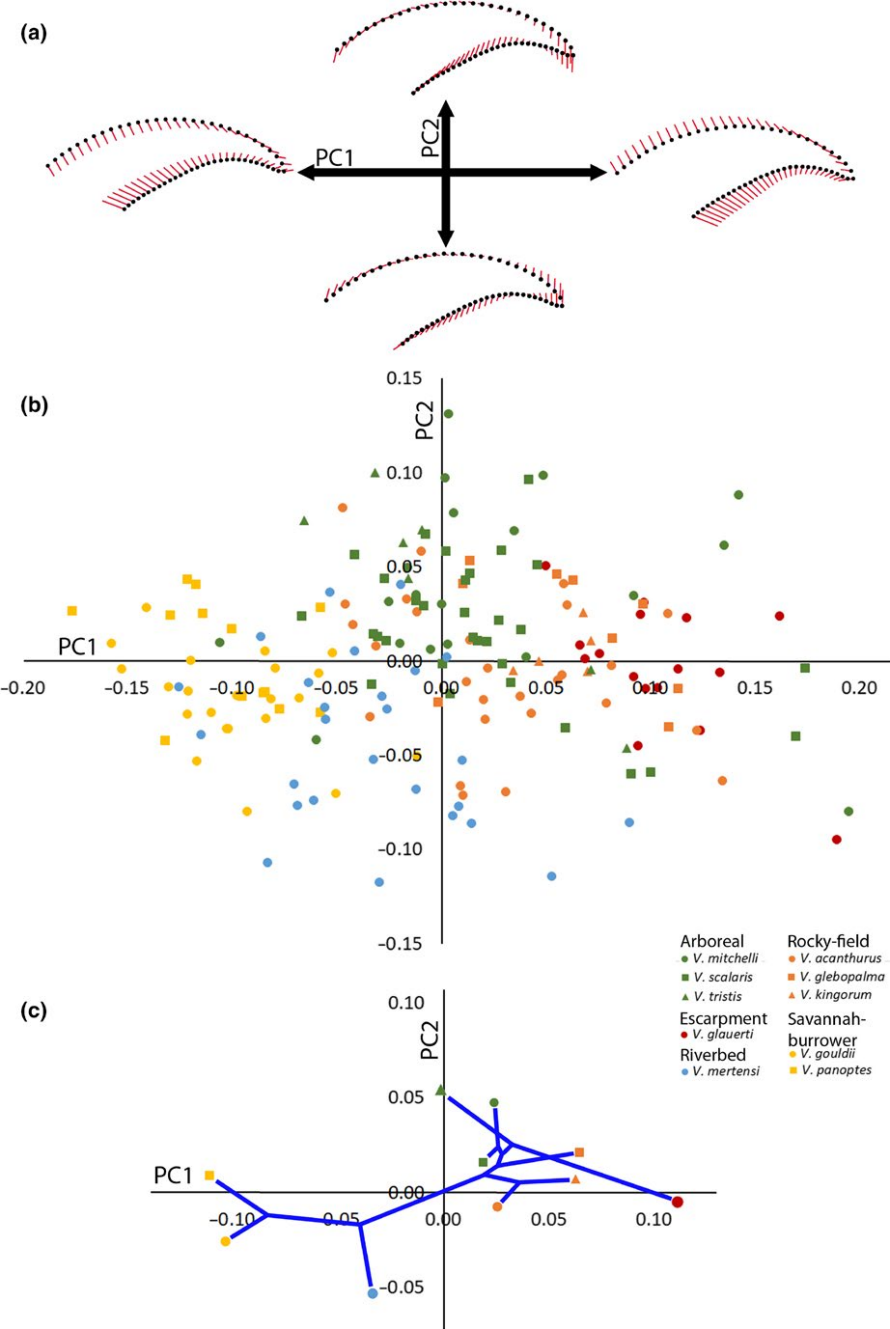
Principal components analysis for all claws. (a) Represents vector diagrams of maximum and minimum shape variance of all varanids for the first and second principal components (PC). Black points indicate the mean claw shape (PC = 0), and red vectors indicate deviation from said mean represented by the most extreme sample along the respective PC axis. (b) Represents principal components scores for all claws in the sample for PC1 and 2. Habitat/locomotor group and species are distinguished by color and shape, respectively. (c) Phylomorphospace of species mean PC scores (based on Thompson et al., 2009)

The first two PCs accounted for >80% of the total shape variance. PC1 (61.62%) displayed variation in claw height relative to length. Short, high claws were indicated by positive values, and low, elongate ones as negative (Figure 5a). PC2 (20.87%) could be defined as claw curvature. Positive values defined more curved claws and negative values indicated less curved claws. Curved claws also reflected a narrowing at the tip.

Savannah‐burrower varanids had some of the most negative PC1 values, and surrounded the mean of PC2 (Figure 5b). Opposite them was the Escarpment species with all positive PC1 scores. Arboreal varanids surrounded the mean of PC1, with majority possessing positive PC2 scores. Riverbed varanids had average to negative PC values, with some of the most negative PC2 values in the sample. The Rocky‐field scores rested between Arboreal and Escarpment, and overlapped both.

The phylomorphospace (Figure 5c) clustered the Gouldii clade together with average to negative PC1 and 2 values, and Odatria primarily in the opposite quadrant with average to positive PC1 and 2 values. *Varanus gouldii* and *V. panoptes* clustered with low PC1 scores, reflecting long claws with shallow curves and low heights. *Varanus mertensi* separated from them with a low PC2 score, as its claws were uncurved. Within the Odatria, *V. glauerti* stood out with a high PC1 score that displayed a very short and high claw. *Varanus tristis* had the highest PC2, indicating its claws both curved and tapered to a distinct point. The remainder of the Odatria clustered together.

## 4 DISCUSSION

### 4.1 Summary and hypotheses

There were significant and noticeable differences between the claw morphology of each Habitat/substrate group. Therefore, our primary hypothesis that claw morphology correlated with substrate use was supported. The claws of Riverbed species were long and straight. The Savannah‐burrower species were even longer, slightly curved, and relatively large. Arboreal, Rocky‐field, and Escarpment groups had claws that were all relatively high, with varied curvature. On average, Escarpment had the highest and shortest claws, and Arboreal had the most curved claws with a distinct “pointed” tip. The Rocky‐field group overlapped both these groups, with means rested in‐between. Allometry and phylogeny influenced claws as well, as Odatria were all relatively small climbers and Gouldii were both large and terrestrial.

### 4.2 Habitat‐related explanations for claw morphology

The claws of the Kimberley varanid guild shared striking similarities with those of previously studied taxa, especially other lizards and birds. Because we did not directly measure performance here, our conclusions about the influence of habitat on claw morphology were based on analogy with previously studied taxa.

Greater claw height and curvature are considered indicative of climbers (Crandell et al., 2014; Ribas et al., 2004; Tulli et al., 2009), and especially true for mostly vertical climbers (Glen & Bennett, 2007). It is not surprising that the Arboreal, Rocky‐field, and Escarpment taxa collectively reflected a similar condition, considering accounts of some sort of climbing existed for all species (see section 2.1). Claws interlock with substrate when climbing, thereby generating nonvertical contact surfaces (Biewener, 2003). These surfaces are typically perpendicular to adductor forces, resulting in a vertical reaction force supporting the animal (Cartmill, 1985). A relatively high claw has a mechanical advantage, and can withstand these forces as the animal clings to substrate (Alexander, 1968). The curved, narrow‐tipped Arboreal claws were specialized for creating these contact surfaces by penetrating the wood and bark they often climb (Biewener, 2003). The Escarpment claws were significantly shorter, and consequently higher, than those of their arboreal relatives. Two possible explanations exist for why this is. First, this particular claw morph may be specialized to deal with rough substrates (Zani, 2000) such as sandstone. These surfaces cannot be penetrated like wood, and the claw must therefore interlock with sand‐size particles when climbing. Having long claws would project the varanid's center of mass away from its own supports, increasing its likelihood of toppling (Cartmill, 1985). An alternative explanation is that the hard sandstone substrate could have worn away part of the claw. Constantly climbing the escarpment may have grinded down the tip, giving the claw a shorter, less curved appearance. There was little evidence of claw wear apparent to the naked eye, so we feel the former is more likely.

Members of the Rocky‐field group may be considered locomotor generalists, as accounts indicate they both free‐roam and rock‐climb. *Varanus glebopalma* is especially proficient in both behaviors (Sweet, 2004b); it habitually rushes after prey, sprints across boulder fields, and ascends vertical rock faces. Rocky substrate was therefore reflected in claw shape; claws were relatively short and high, similar to (although to a lesser degree than) Escarpment taxa. The Rocky‐field morphotype might allow these varanids to interact with rocky substrate in a more versatile way than *V. glauerti*, which is considered to be “wholly saxicolous” in the Kimberley (Sweet, 2004a p.369).

The Savannah‐burrower and Riverbed claws reflected terrestrial locomotion primarily. A shallow‐curved claw would be ideal for providing grip as the foot is rotated against substrate when running, as seen in ground birds (Birn‐Jeffery et al., 2012; Feduccia, 1993; Glen & Bennett, 2007; Pike & Maitland, 2004). Both biomechanical models and empirical studies correlate relative limb lengths and speed (Garland, 1985; Losos, 1990b; Sinervo & Losos, 1991), because increasing stride length will transport the animal farther with each step (Biewener, 2003). Replacing claw height with length would increase overall limb length and consequently sprint speed (Teixeira‐Filho et al., 2001; Tulli et al., 2009, 2012), and the Savannah‐burrower varanids are known to have especially high sprint speeds (Clemente, Withers, & Thompson, 2012; Clemente et al., 2009).

The need to loosen and move resistant material is the main obstacle of a digging vertebrate (Hildebrand, 1985). This requires much force, and the digging tool must be modified to resist said forces. Vertebrates that “scratch‐dig” extensively tend to have long, and disproportionately large, claws (Bramble, 1982; Lautenschlager, 2014; Taylor, 1978; Warner, Tucker, Filoramo, & Towey, 2006). The Savannah‐burrower taxa share this trait, as they are arguably the most proficient diggers of the Kimberley. Both species maintain communal warrens consisting of multiple burrows (Christian, 2004b). *Varanus gouldii* can dig several burrows up to 5 meters long in sequence to excavate invertebrates (Thompson, 2004; Whitford, 1998). *Varanus panoptes* produces a complex spiraling burrow, the deepest reptile nest sites on record (Doody, James, Colyvas, McHenry, & Clulow, 2015; Doody et al., 2018).

The degree to which other Kimberley taxa dig is either much less extreme, or unknown (Husband, 1980). This is limited to the shallow excavation of tree hollows (Sweet, 2004a; Thompson & Pianka, 1999), termite mounds (Smith et al., 2004), and turtle egg nests (Christian, 2004a; Kennett et al., 1993). Although of similar body size, *V. mertensi* nests are much shallower and simpler than *V. gouldii* or *V. panoptes* (Rhind et al., 2016), so there is less selection pressure to enlarge or elongate claws.

### 4.3 Nonhabitat‐related explanations for claw morphology

It was not surprising that phylogenetic signaling plays a significant role in varanid claw morphology, considering it also strongly influences claw shape in other lizards (Tulli et al., 2009, 2011, 2012) and birds (Birn‐Jeffery et al., 2012; Fowler et al., 2009). We could not develop rigorous phylogenetic conclusions about Australian varanids as a whole, but tentative inferences may be drawn. High PICs tended to link with the establishment of new Habitat/substrate groups within phylogenetic history. The ancestral state of Odatria was likely a high claw for climbing, which became only slightly more specialized to cope with the Arboreal and Rocky‐field conditions. *Varanus glauerti* went through a substantial morphological change when it transitioned to vertical sandstone. A relatively long claw may have been the ancestral condition of the Gouldii group. The Savannah‐burrower morphotype appeared after the crown group separated from the *V. mertensi* line, when they adopted their digging‐heavy lifestyle.

The positive size allometry apparent in claws has also been seen in other locomotor structures such as limbs (Christian & Garland, 1996). This is most likely a consequence of the cubic scaling of mass with size increase (Alexander, 1985; McMahon, 1973), as mass is highly correlated with SVL in varanids (Thompson, 2004). Claws can bear much of the weight of the animal when running and/or climbing, and, as a varanid's body size increases, mass increases at a greater rate than claw cross‐sectional area. Therefore, an allometric increase in relative claw size would compensate for this. The fact that shape allometry was heavily influenced by Habitat/substrate group may be an indicator of how body size limits locomotor mode in Varanidae. Large size could be detrimental to a climbing animal, as this increases the likelihood of structural failure associated with falling (Biewener, 1982; Cartmill, 1985). The climbing varanids were limited to ≤340 mm SVL, which the terrestrial species surpassed. This resulted in a general correlation between climbing/terrestrial claw morphotypes and body size. Other monitor lizards have shown a similar connection, as *Varanus komodoensis* changes from climbing to terrestrial locomotion as it grows larger (Auffenberg, 1981). This was not the case in the Kimberley taxa, as no ontogenetic changes in claw shape within species were exhibited.

### 4.4 Claw morphology and Niche partitioning in the Kimberley

The fact that claw morphology correlated well with function suggests that claws may play a role in niche separation in the Kimberley varanid guild. Competition between sympatric varanid species may be reduced by maintaining disparate foraging locales (Pianka, 1994), and we have shown certain claw morphotypes to be ideal for particular habitats. This may function to isolate guild members from one another and allow for coexistence. Claws may allow the Arboreal taxa to physically separate themselves from the larger monitors of the savannah by living in trees, eliminating them as competition and potential predators. On the ground, the specialized claws of the Savannah‐burrowing taxa allowed them to exploit the terrestrial prey by chasing after and/or excavating them. This claw morphotype limits their climbing of trees though. It also constrains them to the savannah, as rocky terrain would be ill‐suited for burrowing. *Varanus glauerti* is the only varanid typically found along vertical escarpments in the Kimberley gorges, so its uniquely shaped claws allow it to solely exploit this habitat.

Varanids often use body size differences (Farlow & Planka, 2002) and behavior to faciltate niche separation. These are probably major driving forces in the Kimberley, especially in taxa with similar claw morphologies. *Varanus mitchelli* is associated with rivers where it eats fish (Losos & Greene, 1988; Schultz & Doody, 2004; Shine, 1986), isolating it from the other, less riparian, arboreal taxa. This diet also separates it from *V. mertensi*, which specializes in crustaceans and turtle eggs (Losos & Greene, 1988; Shine, 1986). *Varanus panoptes* may separate itself from *V. gouldii* in that it is typically larger (Christian, 2004b; Thompson, 2004), and incorporates more riparian‐zone prey (Pianka, 1994; Shine, 1986; Thompson, 1996). Rocky‐field monitors almost certainly partition themselves through size variation, as this correlates with their diet. *Varanus kingorum* is the smallest and limited to eating small arthropods, whereas *V. glebopalma* consumes mostly vertebrates (James, Losos, & King, 1992; King, 2004). *Varanus glebopalma* is also the only monitor known to feed at dusk (Rhind et al., 2013; Swanson, 1979).

## 5 CONCLUSIONS

Our results showed claw morphology was highly variable in the Kimberley monitor lizards, and correlated well with substrates found in their respective habitats as well as locomotor behaviors. This makes claws likely ecomorphological candidates for niche separation in this predator guild, especially when considered in tandem with body size and habitat selection behaviors. Guilds are often defined by shared trophic resources (Simberloff & Dayan, 1991), and varanids typically have intersecting diets due to their opportunistic feeding strategies (example in Sutherland, 2011). Feeding structures such as skulls and teeth would be potentially poor ecomorphological characters for differentiating niche, as such traits have linked particularly well with diet in monitor lizards (Rieppel & Labhardt, 1979; D'Amore, 2015, unpublished data). We suggest that morphological traits associated with locomotion, such as claws, may be more reliable candidates for niche partitioning in these situations, as they link to the occupation of certain habitats.

A major limitation of our study is the fact we only considered two dimensions, and morphological variation along the medial‐lateral axis almost certainly influences function. For example, the degree of taper along this axis could affect interaction with substrate. Claws built for puncturing wood would benefit from a very narrow tip (Cartmill, 1985), whereas burrowers may benefit from a wide claw as it would help transport soil (Hildebrand, 1985). Excluding the third dimension also prohibits investigating certain biomechanical principles by assessing lateral bending strengths (performed for teeth by Valkenburgh and Ruff (1987)), or conducting finite element modelling (Lautenschlager, 2014; Manning et al., 2009). Future studies should consider in what way medial‐lateral claw characters may influence claw function.

In addition to broader applications, several surveys and testable hypotheses may be developed from our work. Admittedly our assessment of claw function is correlative, as claw performance has yet to be directly tested in these varanids. Performance studies are therefore necessary to confirm our assertions about the functional significance of these claws (*sensu* Wainwright, 1991; Irschick, 2002). This should then be followed by investigations into how claws specifically influence patterns of resource use in the Kimberley. Although stomach content studies exist for these monitors, more studies investigating diet and prey capture methods would allow for elaboration on habitat use. Habitat/substrate groups may be expanded across most Australian varanids to see if claw shape varies to such a degree in other guilds, as well as determine the extent that phylogenetic signal plays a role in claw morphology.

## CONFLICT OF INTEREST

None declared.

## AUTHOR CONTRIBUTIONS

DD, SC, and CM conceived the ideas and designed methodology; DD, SC, SD, DR, and CM collected the data; DD analyzed the data and led the writing of the manuscript.

## DATA ACCESSIBILITY

Data has been uploaded to Dryad (https://doi.org/10.5061/dryad.cf2hs41).
